# Risk factors of different mortality periods in older patients with end-stage renal disease undergoing urgent-start peritoneal dialysis: a retrospective observational study

**DOI:** 10.1186/s12877-024-04931-4

**Published:** 2024-04-15

**Authors:** Shizheng Guo, Liming Yang, Xueyan Zhu, Xiaoxuan Zhang, Zhanshan Sun, Lingfei Meng, Yangwei Wang, Jian Li, Siyu Cheng, Xiaohua Zhuang, Wenpeng Cui

**Affiliations:** 1 Department of Nephrology, the Second Bethune Hospital of Jilin University, 130022 Changchun, Jilin Province China; 2 Department of Nephrology, the First Bethune Hospital of Jilin University-the Eastern Division, 130021 Changchun, Jilin Province China; 3 Department of Nephrology, Jilin Central Hospital, 132011 Jilin, Jilin Province China; 4 Department of Nephrology, Jilin Province FAW General Hospital, 130013 Changchun, Jilin Province China; 5 Department of Nephrology, Xing’an League People’s Hospital, Ulan Hot, 137499 Inner Mongolia Autonomous Region, China

**Keywords:** Urgent-start dialysis, Elderly, Six months, Analysis of death

## Abstract

**Background:**

The first six months of therapy represents a high-risk period for peritoneal dialysis (PD) failure. The risk of death in the first six months is higher for older patients treated with urgent-start PD (USPD). However, there are still gaps in research on mortality and risk factors for death in this particular group of patients. We aimed to investigate mortality rates and risk factors for death in older patients with end-stage renal disease (ESRD) receiving USPD within and after six months of therapy.

**Methods:**

We retrospectively studied the clinical information of older adults aged ≥ 65 years with ESRD who received USPD between 2013 and 2019 in five Chinese hospitals. Patients were followed up to June 30, 2020. The mortality and risk factors for death in the first six months of USPD treatment and beyond were analyzed.

**Results:**

Of the 379 elderly patients in the study, 130 died over the study period. During the follow-up period, the highest number (45, 34.6%) of deaths occurred within the first six months. Cardiovascular disease was the most common cause of death. The baseline New York Heart Association (NYHA) class III–IV cardiac function [hazard ratio (HR) = 2.457, 95% confidence interval (CI): 1.200–5.030, *p* = 0.014] and higher white blood cell (WBC) count (HR = 1.082, 95% CI: 1.021–1.147, *p* = 0.008) increased the mortality risk within six months of USPD. The baseline NYHA class III–IV cardiac function (HR = 1.945, 95% CI: 1.149–3.294, *p* = 0.013), lower WBC count (HR = 0.917, 95% CI: 0.845–0.996, *p* = 0.040), lower potassium levels (HR = 0.584, 95% CI: 0.429–0.796, *p* = 0.001), and higher calcium levels (HR = 2.160, 95% CI: 1.025–4.554, *p* = 0.043) increased the mortality risk after six months of USPD.

**Conclusion:**

Different risk factors correlated with mortality in older adults with ESRD within and after six months of undergoing USPD, including baseline NYHA class III–IV cardiac function, WBC count, potassium, and calcium levels.

## Background

End-stage renal disease (ESRD) poses a major global public health threat. The number of patients using renal replacement therapy has been estimated to at least double globally by 2030, with the most rapid increase occurring in Asia [1]. Due to complicating factors such as late referrals and unforeseen deterioration of renal function, many patients require an urgent start of dialysis treatment [2]. Although peritoneal dialysis (PD) has unique advantages, such as hemodynamic stability, protection of residual kidney function, no need for vascular access, and lower technical requirements [3, 4], urgent-start hemodialysis (USHD) is preferred as the first option in most cases [5]. However, although fewer patients are treated with urgent-start peritoneal dialysis (USPD) than with USHD, many studies have suggested that the former is also a viable and well-tolerated option [4, 6, 7]. 

With increasing life expectancy, the demand for dialysis in older patients is rising [8, 9]. Patients above 65 years of age undergoing dialysis account for approximately 28.85% of the total dialysis population in China and have the highest mortality rate, estimated at 91.69/1000 patient-years [10]. Although older adults may present more intestinal complications, comorbid conditions, physical decline, impaired mobility, or cognitive decline [11], recent studies still support the use of USPD in this particular age group [12, 13]. Nonetheless, few studies are available evaluating older patients treated with USPD. Thus, an analysis of mortality and associated risk factors in this population is lacking. A retrospective study from China analyzed the factors associated with mortality among older patients receiving urgent-start dialysis; however, risk factors related to older patients treated exclusively with USPD were not investigated [13]. 

Several studies have reported that the first six months of therapy represents a high-risk period for PD failure [14, 15]. Thus, for older patients, the first six months after placement may represent an even higher risk period. Presumably, the mortality and risk factors for death within and after six months of USPD also differ. Considering that PD may represent life-long renal replacement treatment, it is necessary to determine the short- and long-term mortality risks.

We conducted this study to analyze the mortality rates of older adults with ESRD who were treated with USPD, to explore potential risk factors associated with all-cause mortality at different treatment periods, and to predict high-risk groups to allow timely intervention and potentially modify any reversible risk factors and further improve the survival of these patients.

## Materials and methods

### Patients

We retrospectively reviewed records from all patients diagnosed with ESRD who received PD catheter insertion and were treated with USPD between 1 January 2013 and 31 December 2019 at the following five participating centers: the Second Bethune Hospital of Jilin University, the Eastern Division of the First Bethune Hospital of Jilin University, the Jilin Central Hospital, Jilin Province FAW General Hospital, and the Xing’an League People’s Hospital. Patients who started PD therapy within 14 days of catheter placement were considered as having received USPD therapy [9, 16, 17]. Regular PD was defined as the initiation of PD therapy 2 weeks after placement of the catheter. Patients aged < 18 years, those with incomplete clinical data, and those with uncertain time of death were excluded from this study. Follow-up was completed on 30 June 2020 or when patients experienced death, technical failure, or kidney transplantation. Informed consent was not required given the retrospective nature of the study. This study complied with the requirements of the Declaration of Helsinki. The Ethics Committee of the Second Norman Bethune Hospital of Jilin University authorized the study design (approval number: 2,020,026).

### Condition and care before dialysis

Before entering dialysis, we provided educational information to each outpatient with chronic kidney disease, including requesting regular follow-up every 1–3 months for better management of blood pressure, blood glucose, proteinuria, and other markers, avoidance of exertion, infections, and other factors that may exacerbate renal damage, dietary counseling, and evaluation of the timing of dialysis; and dialysis modality education for inpatients was provided in advance for co-determination of dialysis modality. The main indications we chose for USPD were symptoms of uremia (for example, nausea, vomiting, or manifestations of encephalopathy), edema or pulmonary edema that was difficult to correct with conservative therapy, hyperkalemia (K > 6.5 mmol/L), and acidosis (serum bicarbonate < 10 mEq/L) [18]. Abdominal or cardiothoracic surgery within 30 days, abdominal infections, severe respiratory insufficiency, life-threatening volume overload, acidosis, and hyperkalemia were contraindications to USPD [19]. Before deciding on the dialysis modality at each center, the specialist informed the patient and the patient’s family about the advantages and disadvantages of PD and HD and helped them to make a better choice based on their situation.

### Treatment procedure

All patients received skin preparation, prophylactic antibiotics, and bowel and bladder emptying before the operation. All PD catheters were inserted anatomically or via puncture by physicians with extensive experience in catheter placement. PD was initiated within two weeks after insertion [20]. Training and education regarding PD were provided by experienced physicians and nurses before patients were discharged to ensure that both patients and caregivers could complete the procedure successfully and independently. The main modes of dialysis were automated PD (APD) and continuous ambulatory PD (CAPD). Dialysis was initiated in the format of low-exchange capacity in the supine position. The initial exchange volume was 0.5–1.0 L, steadily increasing to 2 L over two weeks depending on patients’ symptoms and examination indicators. Patients receiving CAPD therapy underwent three to four exchanges daily, and those receiving APD therapy underwent six to nine exchanges daily. A glucose-based dialysate (Guangzhou Baxter Healthcare Corporation, Guangzhou, Guangdong Province, China, or Huaren Pharmaceutical, Qingdao, Shandong Province, China) was used as the routine dialysate, and the specific dialysis prescription was determined based on the patient’s condition by the attending physicians. All patients were advised to perform the peritoneal equilibration test every three to six months to assess peritoneal function and dialysis adequacy. The dialysis regimen was adjusted partially based on the results, with a goal of Kt/V urea ≥ 1.70 and creatinine clearance ≥ 50 L/week/1.73 m^2^.

### Data collection

Clinical data was collected through the medical records system of each participating center and included demographic information, laboratory indices, clinical outcome, time of death, and cause of death. Demographic information consisted of sex, age, date of catheter placement, primary disease, comorbidity (hypertension and diabetes), cardiac function classification, and history of abdominal surgery. The baseline laboratory indices consisted of pre-operative white blood cell (WBC) count, hemoglobin, albumin, estimated glomerular filtration rate (eGFR), potassium, calcium, and phosphorus levels. Clinical outcomes included continued PD, death, technical failure, kidney transplantation, or loss of follow-up. Technical failure was regarded as the transition to hemodialysis for more than one month [21]. Cardiovascular diseases (CVD) included arrhythmias, cardiac arrest, acute myocardial infarction, congestive heart failure, cerebrovascular accident, or peripheral vascular disease [22]. Based on the New York Heart Association (NYHA) classification, cardiac function was classified into five levels: class 0 for no symptoms of heart failure; class I for no restriction of daily activities; class II for mild restriction of daily activities; class III for moderate restriction of daily activities; and class IV for resting symptoms of heart failure [23]. 

### Statistical analysis

For qualitative variables, data were described by frequencies and percentages (n, %), and chi-square tests were used to compare different periods. For quantitative variables, normally distributed data were reported by mean ± standard deviation ($\stackrel{-}{x}$± s), and t-tests were utilized to compare groups. In addition, non-normally distributed data were reported by the interquartile range [M (25, 75)], and rank sum tests were utilized to compare groups. The Kaplan–Meier curve was constructed to describe population survival and the Log–rank test was performed to determine disparities in survival across patients with different grades of cardiac function. Cox regression models were constructed to explore independent factors related to all-cause mortality. Variables with *p*-values < 0.05 in the univariate analysis and variables that might affect mortality (serum potassium and calcium for the model within 6 months and WBC count for the model after 6 months) were corrected in the multivariate COX model. Differences with *p*-values < 0.05 were considered statistically significant. All figures were plotted using GraphPad Prism version 8.0 (GraphPad Software, San Diego, CA, USA). All data were analyzed using SPSS version 26.0 (IBM Corp., Armonk, NY, USA).

## Results

### Mortality rates and cause of death

**Fig. 1:**
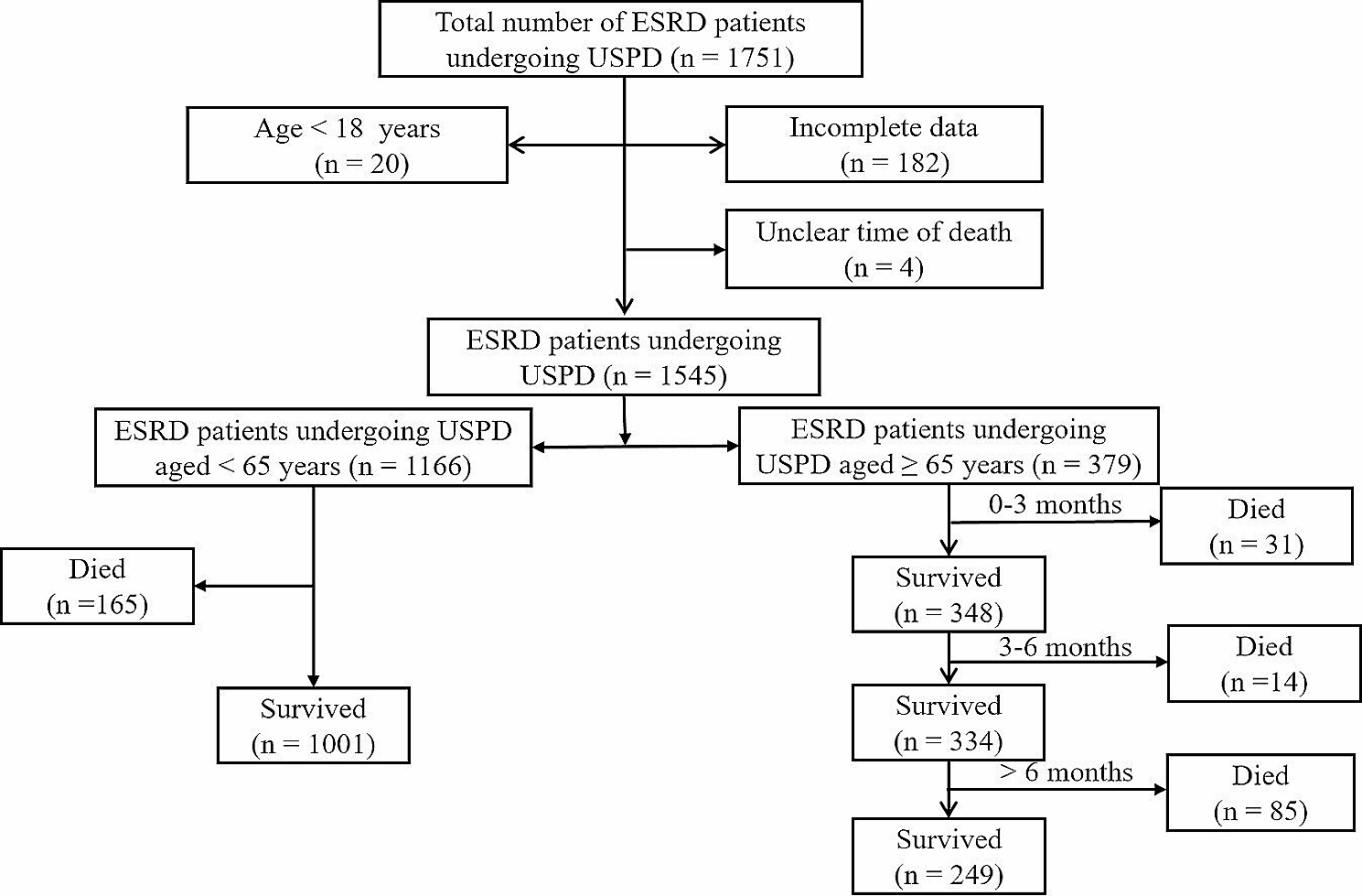
Flow chart

**Fig. 2:**
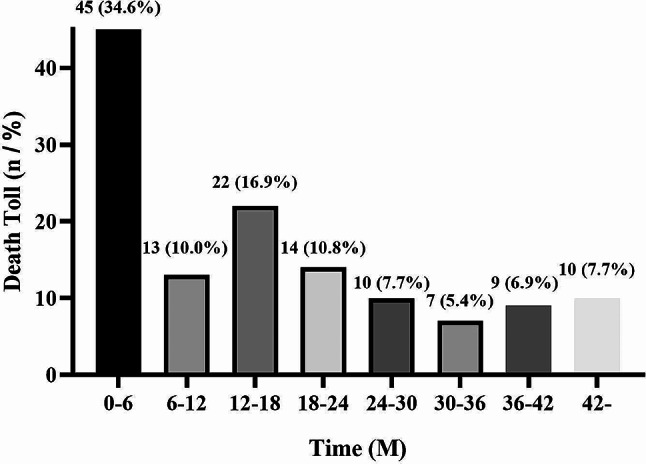
Survival curves of the older patients and younger patients undergoing USPD

**Fig. 3:**
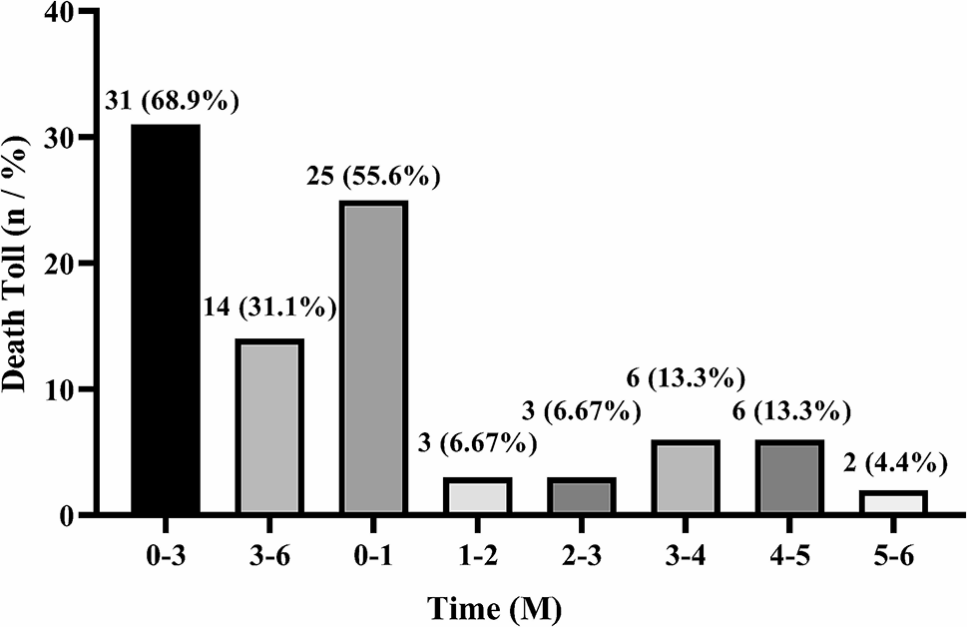
**a** Mortalities among older patients undergoing USPD in different periods during the whole follow-up period. **b** Mortalities among older patients undergoing USPD in different periods during the first six months

A total of 1751 individuals received USPD over seven years, and 1166 patients aged < 65 years and 379 patients aged ≥ 65 years were enrolled in our study (Fig. 1). Throughout the follow-up period, 165 younger patients and 130 elderly patients died. Figure 2 shows the cumulative survival of 379 older patients and 1166 younger patients throughout the follow-up period. There was a significant difference in survival between the two groups (*p* < 0.05). The 1-month, 3-month, 6-month, 1-year, 2-year, and 3-year survival rates in the elderly patient population were 93.4%, 91.8%, 88.3%, 84.3%, 73.0%, and 65.9%, respectively. Survival rates for the corresponding periods in the younger patient population were 98.9%, 98.1%, 97.2%, 94.0%, 89.7%, and 84.7%, respectively. When we analyzed mortality in elderly patients, we observed that the first six months of USPD therapy was the period with the highest number of deaths (45, 34.6%), with a mortality rate of 11.9% (Fig. 3a). Furthermore, deaths in the first month accounted for more than half of the deaths occurring in the first six-month period (25, 55.6%) with a mortality rate of 7.0%, and 31 died in the first three months, accounting for 68.9% of the total number of deaths, with a mortality rate of 8.2% (Fig. 3b). Regarding the causes of death, the top three causes of death in each of the periods presented in Table 1 were cardiovascular diseases, multiple organ failure, and infections. Among these, cardiovascular disease is firmly in the top position as the leading cause of death, with a high rate of about 30%.

**Table 1:** Distribution of the cause of death in older patients treated with USPD

Cause of death	0-3 M (n, %)	3-6 M (n, %)	> 6 M (n, %)
Total	31 (100)	14 (100)	85 (100)
Cardiovascular diseases	11 (35.5)	5 (35.7)	24 (28.2)
Multiple organ failure	7 (22.6)	2 (14.3)	9 (10.6)
Infections	1 (3.23)	1 (7.14)	9 (10.6)
Malignancy	1 (3.23)	0 (0)	5 (5.9)
Respiratory failure	4 (12.9)	0 (0)	2 (2.35)
Unknown	6 (19.4)	6 (42.9)	34 (40.0)
Others	1 (3.23)	0 (0)	2 (2.4)

*Abbreviation* USPD, urgent-start peritoneal dialysis

### Clinical characteristics

We compared the clinical features and baseline data of younger patients and elderly patients as well as elderly patients who died during the first six months of USPD treatment and beyond with those who survived the same period. Compared to younger patients, older patients have a lower proportion of men, a higher proportion of comorbid diabetes, poorer cardiac function, lower levels of blood albumin, and lower levels of serum phosphorus (*p* < 0.05) (Table 2). Compared to survivors, patients who died within the first six months presented with worse baseline cardiac function, lower serum albumin levels, and higher WBC counts (*p* < 0.05) (Table 3). Patients who died after six months presented with lower baseline WBC count, serum potassium levels, and serum phosphorus levels than those of patients who survived (*p* < 0.05) (Table 4).

**Table 2:** Characteristics and baseline data of elderly and younger patients treated with USPD

Variables	Younger Patients (*n* = 1166)	Older patients (*n* = 379)	*t/χ* ^*2*^ */Z*	*p*-value
Male (n, %)	688 (60.2)	173 (45.6)	24.690	0.000
Etiology (n, %)			14.159	0.015
Glomerulonephritis	442 (38.7)	123 (32.5)		
Diabetic nephropathy	331 (29.0)	124 (32.7)		
Hypertensive nephropathy	176 (15.4)	66 (17.4)		
Interstitial nephritis	29 (2.1)	6 (1.6)		
Polycystic kidney	24 (2.1)	6 (1.6)		
Others	140 (12.3)	37 (10.3)		
Co-morbidities (n, %)				
Hypertension	1051 (92.0)	345 (91.0)	0.379	0.538
Diabetes	314 (34.5)	167 (44.1)	11.177	0.001
NYHA-FC (n, %)			65.042	0.000
0	722 (63.2)	150 (39.6)		
I–II	238 (20.8)	129 (34.0)		
III–IV	182 (15.9)	100 (26.4)		
Abdominal surgery history (n, %)	133 (11.6)	55 (14.5)	2.157	0.142
Laboratory Index				
WBC (*10^9^/L)	6.90 (5.35, 8.16)	6.93 (5.27, 8.28)	-1.084	0.278
Hb (g/L)	84.00 (72.00, 97.00)	84.83 (73.00, 95.00)	-0.059	0.953
Alb (g/L)	34.34 (30.68, 38.00)	33.50 (30.30, 36.27)	-2.451	0.014
eGFR (ml/min/1.73m^2^)	5.30 (3.97, 7.10)	5.36 (4.17, 6.96)	-0.514	0.607
K (mmol/L)	4,61 (4.02, 5.06)	4.60 (4.02, 5.18)	-0.089	0.929
Ca (mmol/L)	1.96 (1.79, 2.12)	1.97 (1.82, 2.14)	-0.792	0.428
P (mmol/L)	2.01 (1.62, 2.36)	1.83 (1.42, 2.14)	-5.271	0.000

*Abbreviations* USPD, urgent-start peritoneal dialysis; NYHA-FC, New York Heart Association functional classification; WBC, white blood cell; Hb, hemoglobin; Alb, albumin; eGFR, estimated glomerular filtration rate; K, serum potassium; Ca, serum calcium; P, serum phosphorus

**Table 3:** Characteristics and baseline data of older patients treated with USPD within six months

Variables	Died (*n* = 45)	Survived (*n* = 334)	*t/χ* ^*2*^ */Z*	*p*-value
Age (years)	73.33 ± 5.95	71.76 ± 5.71	-1.723	0.086
Male (n, %)	22 (48.9)	151 (45.2)	0.216	0.642
Etiology (n, %)	-	-	3.181	0.671
Glomerulonephritis	12 (26.7)	111 (33.2)	-	-
Diabetic nephropathy	17 (37.8)	107 (32.0)	-	-
Hypertensive nephropathy	10 (22.2)	56 (16.8)	-	-
Interstitial nephritis	3 (6.7)	18 (5.4)	-	-
Polycystic kidney	0 (0.0)	6 (1.8)	-	-
Others	3 (6.7)	36 (10.8)	-	-
Co-morbidities (n, %)				
Hypertension	40 (88.9)	305 (91.3)	0.286	0.593
Diabetes	24 (53.3)	143 (42.8)	1.780	0.182
NYHA-FC (n, %)	-	-	3.885	0.048
0	12 (26.7)	138 (41.3)	-	-
I–II	13 (28.9)	112 (33.5)	-	-
III–IV	20 (44.4)	84 (25.1)	-	-
Abdominal surgery history (n, %)	6 (13.3)	49 (14.7)	0.057	0.811
Laboratory Index				
WBC (*10^9^/L)	8.28 (5.69, 11.18)	6.60 (5.25, 8.33)	-2.827	0.005
Hb (g/L)	85.00 (74.50, 98.00)	84.00 (72.56, 95.00)	-0.447	0.655
Alb (g/L)	31.40 (29.20, 35.03)	33.68 (30.10, 36.83)	-1.992	0.046
eGFR (ml/min/1.73m2)	5.28 (4.18, 7.05)	5.28 (4.14, 7.04)	-0.396	0.692
K (mmol/L)	4.69 ± 0.99	4.62 ± 0.89	-0.479	0.632
Ca (mmol/L)	1.98 ± 0.37	1.96 ± 0.30	-0.399	0.690
P (mmol/L)	1.83 (1.25, 2.39)	1.80 (1.43, 2.14)	-1.723	0.086

*Abbreviations* USPD, urgent-start peritoneal dialysis; NYHA-FC, New York Heart Association functional classification; WBC, white blood cell; Hb, hemoglobin; Alb, albumin; eGFR, estimated glomerular filtration rate; K, serum potassium; Ca, serum calcium; P, serum phosphorus

**Table 4:** Characteristics and baseline data of older patients treated with USPD after six months

Variables	Died (*n* = 85)	Survived (*n* = 249)	*t/χ* ^*2*^ */Z*	*p*-value
Age (years)	72.31 ± 6.25	71.58 ± 5.51	-0.954	0.342
Male (n, %)	40 (47.1)	111 (44.6)	0.157	0.692
Etiology (n, %)	-	-	8.784	0.118
Glomerulonephritis	23 (27.1)	88 (35.3)	-	-
Diabetic nephropathy	37 (43.5)	70 (28.1)	-	-
Hypertensive nephropathy	12 (14.1)	44 (17.7)	-	-
Interstitial nephritis	5 (5.9)	13 (5.2)	-	-
Polycystic kidney	0 (0.0)	6 (2.4)	-	-
Others	8 (9.4)	28 (11.2)	-	-
Comorbidities (n, %)				
Hypertension	80 (94.1)	225 (90.4)	1.128	0.288
Diabetes	43 (50.6)	100 (40.2)	2.814	0.093
NYHA-FC (n, %)	-	-	3.881	0.275
0	31 (36.5)	107 (43.0)	-	-
I–II	26 (30.6)	86 (34.5)	-	-
III–IV	28 (32.9)	56 (22.5)	-	-
Abdominal surgery history (n, %)	13 (15.3)	36 (14.5)	0.035	0.851
Laboratory Index				
WBC (*10^9^/L)	6.63 ± 2.13	7.38 ± 3.64	2.315	0.021
Hb (g/L)	82.00 (71.00, 94.00)	85.00 (73.00, 96.50)	-1.059	0.290
Alb (g/L)	32.93 (30.40, 36.10)	33.98 (30.50, 37.00)	-0.721	0.471
eGFR (ml/min/1.73m2)	5.94 (4.38, 7.37)	5.94 (4.38, 7.37)	-1.667	0.095
K (mmol/L)	4.03 (4.01, 4.76)	4.67 (4.07, 5.32)	-3.450	0.001
Ca (mmol/L)	2.01 ± 0.32	1.94 ± 0.29	-1.863	0.063
P (mmol/L)	1.68 ± 0.51	1.92 ± 0.66	3.060	0.002

*Abbreviations* USPD, urgent-start peritoneal dialysis; NYHA-FC, New York Heart Association functional classification; WBC, white blood cell; Hb, hemoglobin; Alb, albumin; eGFR, estimated glomerular filtration rate; K, serum potassium; Ca, serum calcium; P, serum phosphorus

### Survival analysis

**Fig. 4:**
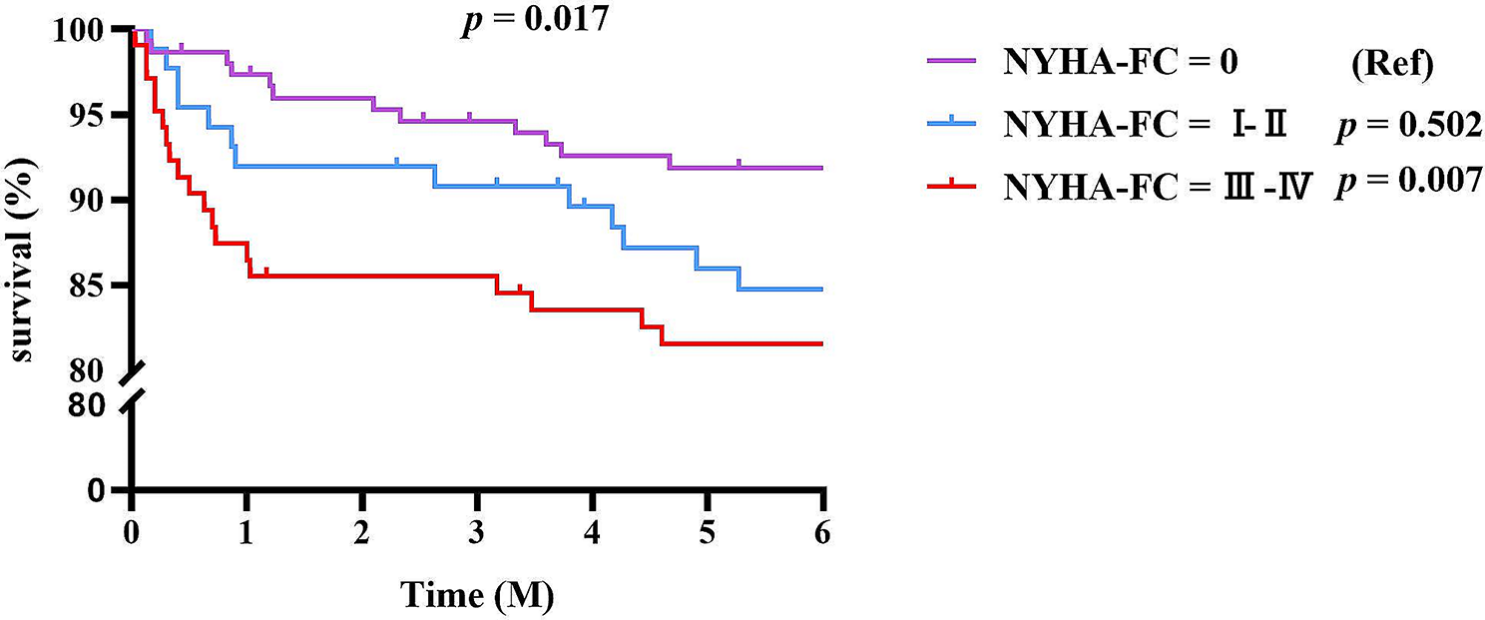
**a** Survival analysis for mortality of older patients receiving USPD according to NYHA-FC within six months **b**: Survival analysis for mortality of older patients receiving USPD according to NYHA-FC after six months

Considering that CVD was the primary cause of mortality, patients were further classified into three groups depending on the NYHA cardiac function classification: class 0, class I–II, and class III–IV for survival analysis. As illustrated in Fig. 4a, individuals with class III–IV cardiac function had worse survival than those without heart failure in the first six months of USPD (*p* = 0.007). As we expected, there was also a statistically significant difference in survival between patients with cardiac function classes III-IV and those with cardiac function class 0 who received USPD for more than 6 months (*p* = 0.033) (Fig. 4b).

### Risk factors for death

**Fig. 5:**
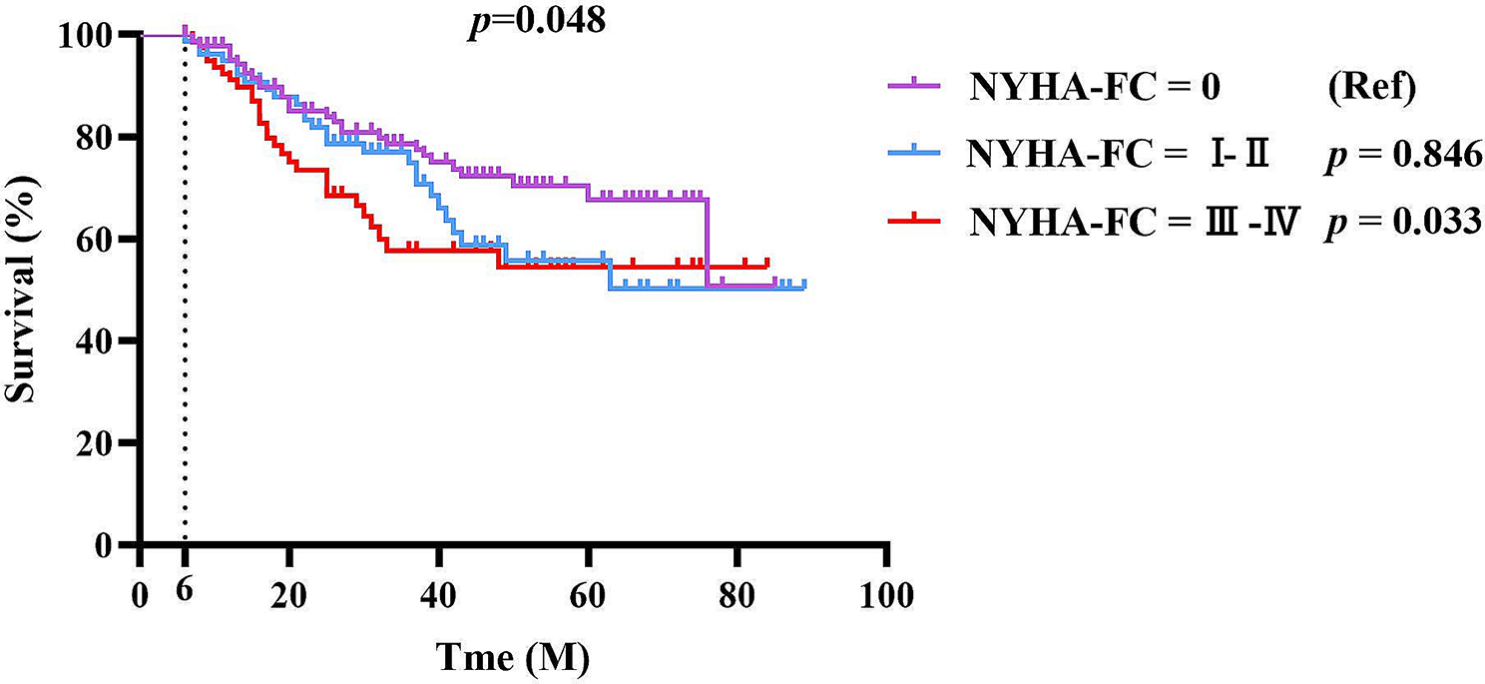
**a**: Risk factors for death in younger patients treated with USPD **b**: Risk factors for death in older patients treated with USPD

We analyzed the risk factors for mortality in the respective overall populations of younger and older patients. Variables with *p* < 0.05 in the univariate COX analysis were included in the multivariate analysis. It was demonstrated in Fig. 5a and b that the risk factors affecting death in the two groups were different. In the younger patient population, older age [hazard ratio (HR) = 1.031, 95% confidence interval (CI): 1.014–1.051, *p* = 0.000], combined diabetes mellitus (HR = 1.532, 95% CI: 1.074–2.166, *p* = 0.018), lower albumin levels (HR = 0.966, 95% CI: 0.939–0.995, *p* = 0.020), lower serum phosphorus levels (HR = 0.757, 95% CI: 0.573–0.999, *p* = 0.000), higher eGFR levels at baseline (HR = 1.058, 95% CI: 1.031–1.085, *p* = 0.000), and cardiac function graded as class III-IV (HR = 1.775, 95% CI: 1.021–2.624, *p* = 0.004) were independent risk factors for death. In the entire elderly patients’ group, only baseline higher serum calcium levels (HR = 1.811, 95% CI: 1.003–3.270, *p* = 0.049) and cardiac function class III-IV (HR = 2.188, 95% CI: 1.440–3.323, *p* = 0.000) were risk factors for death.

**Fig. 6:**
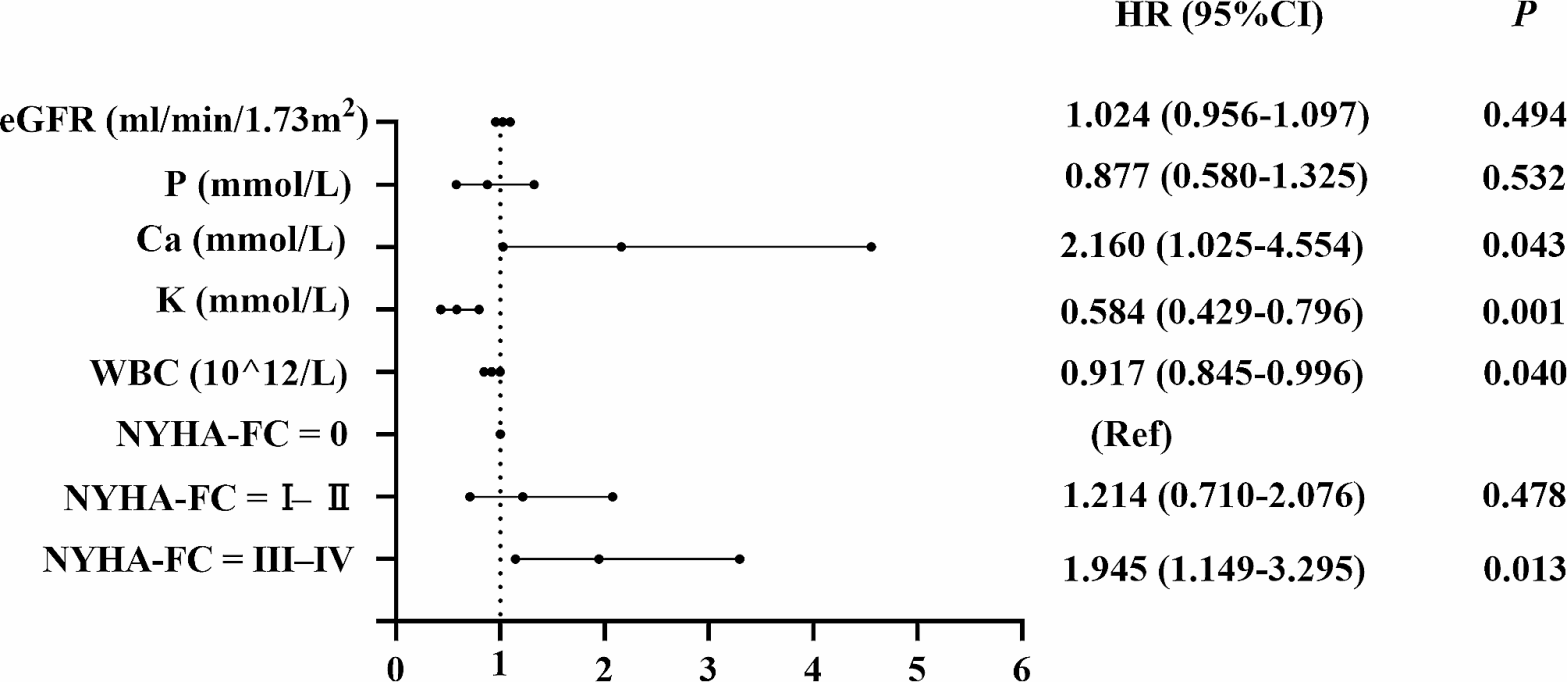
**a** Risk factors for death in older patients treated with USPD within 6 months. **b**: Risk factors for death in older patients treated with USPD after 6 months

Significant factors associated with death in the univariate Cox regression analysis within the first six months were cardiac function classification and WBC count. After correcting for cardiac function classification, WBC count, serum potassium, and calcium in the multivariate analysis (the latter two variables were independent risk factors for death after 6 months, and we wanted to clarify whether they would influence mortality within 6 months), the results suggested that the factors raising the risk of death during the first six months were NYHA class III–IV (HR = 2.457, 95% CI: 1.200–5.030, *p* = 0.014) and higher WBC count (HR = 1.082, 95% CI: 1.021–1.147, *p* = 0.008) (Fig. 6a). Significant variables in the univariate Cox regression analysis after six months were cardiac function classification, eGFR, potassium, calcium, and phosphorus levels. After correcting for cardiac function class, eGFR, potassium, calcium, phosphorus levels, and WBC count in the multivariate analysis (the last variables were independent risk factors for mortality within the first 6 months), risk factors for mortality after six months of USPD were NYHA class III–IV (HR = 1.945, 95% CI: 1.149–3.294, *p* = 0.013), lower potassium levels (HR = 0.584, 95% CI: 0.429–0.796, *p* = 0.001), lower WBC count (HR = 0.917, 95% CI: 0.845–0.996, *p* = 0.040), and higher calcium levels (HR = 2.160, 95% CI: 1.025–4.554, *p* = 0.043) (Fig. 6b).

### Discussion

This was the first multicenter retrospective study to explore risk factors for mortality among older adults with ESRD receiving USPD treatment. Risk factors that significantly influenced mortality in the first six months were baseline class III–IV cardiac function and higher WBC count. Risk factors that significantly influenced mortality after six months were NYHA class III–IV cardiac function, lower WBC count, lower potassium levels, and higher calcium levels at baseline.

We found that the survival rate of elderly patients on USPD was significantly inferior to that of younger patients, which is consistent with the findings of previous studies [24]. At the beginning of dialysis, older patients showed lower levels of albumin and serum phosphorus compared with younger patients, which are important indicators of nutritional status. In addition, the elderly group was more inclined to have comorbid diabetes, which increased the risk of heart failure and cardiovascular events, thus accelerating the death of the patients [25]. Conditions such as malnutrition, the presence of diabetes mellitus, and heart failure contributed to a significantly higher mortality rate in elderly patients with USPD.

In this study, the six-month, one-year, and two-year survival rates of older patients aged 65 years and above receiving USPD were 88.3%, 84.3%, and 73.0%, respectively. Zang et al. from Shanghai noted that the six-month, one-year, and two-year survival rates in older patients treated with USPD were 95.3%, 91.4%, and 86.6%, respectively, reporting higher cumulative survival rates than in our study [13]. As demonstrated in this study, the first six months of USPD treatment, and especially the first month, was the period with the highest proportion of deaths. Zang et al. excluded patients who received dialysis treatment for < 3 months, which might account for the difference in results between the two studies. Because we collected patients with a definite diagnosis of ESRD, patients with less than 3 months of dialysis were not excluded. Additionally, in agreement with the results of other studies [26, 27], our study showed that CVD was the primary cause of death among older adults who underwent USPD, regardless of the period. This is associated with higher rates of malnutrition and diabetes in older patients. Both malnutrition and diabetes contribute to the inflammatory state, causing endothelial cell damage and exacerbating atherosclerosis [26, 28]. Therefore, extra caution given the high risk of cardiovascular mortality is warranted for patients with advanced age.

The risk factor that consistently influenced death in elderly patients throughout the different periods of USPD therapy was baseline cardiac function class III-IV. However, it could be seen that the impact on mortality of this indicator appeared to decrease after 6 months. With dialysis ultrafiltration, the patient’s cardiovascular stability got progressively better, heart failure improved, and patients might die more often from other cardiovascular diseases such as myocardial infarction rather than direct heart failure. Therefore, efforts should be devoted to improving cardiac function at any stage of the management of patients with chronic kidney disease so that the status of cardiac function at baseline at the initiation of dialysis is optimal.

WBC count has been reported to be a significant risk predictor for adverse events in patients undergoing PD [29–31]. One study reported a 23% increase in the risk of death for every 10^9^/L rise in WBC count in an approximate model [32]. The same trend was observed in our model, where the risk of mortality within six months increased by 8% for every 10^9^/L increase in the baseline WBC count in older patients treated with USPD.Interestingly, from the baseline laboratory data, WBC levels were found to be essentially within the normal range. This result is not without clinical relevance. It has been noted that WBC counts in the middle or upper third of the normal range are associated with increased mortality [33], which suggests that WBC counts in the normal range may also offer clinically meaningful information. Higher WBC counts reflect the inflammatory state of the body [32]. Older patients are weaker against infections and more susceptible to sepsis due to decreased immunity and malnutrition [34]. Inflammation and malnutrition are associated with cardiovascular disease among chronic renal failure [35], and all three interact with each other, thereby increasing the risk of death in elderly patients. WBC count is also independently associated with myocardial remodeling and the development of heart failure [36, 37], and thereby negatively affects the survival of patients. However, the fact remains that it was not clear why increased WBC counts raised mortality [32]. We also concluded that lower WBC counts were risk factors for mortality after 6 months. Elderly patients presenting with low WBC count may have bone marrow failure and immunosuppression, resulting in the inability of the organism to respond to some diseases (such as infection, malignancy, or cardiovascular disease) with elevated WBC count [38], which is mainly associated with aging and inflammation in the uremic state.

Lower baseline blood potassium levels in our study as in prior studies were correlated with a higher risk of all-cause death in older patients after six months of USPD [39–41]. Low potassium levels are considered a substitute marker for malnutrition [41], the most likely cause of lower potassium levels might be decreased potassium intake in older patients. After beginning dialysis, and as toxins were gradually removed, the uremic symptoms were gradually alleviated, and the diet somewhat improved. This probably contributed to offsetting the hazard of low baseline potassium levels during the first six months of treatment. Generally, there are additional mechanisms involved in lowering potassium levels after dialysis initiation, such as altered intracellular potassium uptake caused by glucose-containing dialysate and the retained potassium excretion capacity of patients undergoing PD [42]. With increasing age on dialysis, the increased potassium loss may lead to more severe hypokalemia in older patients with pre-existing malnutrition, causing life-threatening arrhythmias and even sudden death. Consequently, in addition to correcting the hypokalemia that is clearly present, it seems also important to correct lower potassium levels (even if they are within the normal range) before starting USPD therapy.

Higher levels of serum calcium, an important contributor to vascular calcification, raise the risk of death among the dialysis population [43, 44]. We reached a similar conclusion that older patients with a higher baseline calcium level were at increased risk of mortality after 6 months of USPD therapy. The occurrence of secondary hyperparathyroidism, exogenous calcium supplementation, use of calcium-containing phosphorus-binding agents, and the possible presence of granulomatous mycosis fungoides (rare) in patients with ESRD contribute to the development of baseline higher calcium level [45, 46], and as PD proceeds, the use of high-calcium dialysate may exacerbate hypercalcemia by increasing the influx of calcium into the extracellular fluid [45]. Some of the above-mentioned factors contributing to increased serum calcium levels and calcification can be corrected by using low-calcium peritoneal dialysate and limiting the usage of calcium-containing phosphate binders [47, 48]. However, whether the above interventions can decrease patient mortality requires further evidence [49]. 

We also analyzed risk factors among younger patients with USPD, and the results were quite different from those of elderly patients. In addition to the well-known risk factors of lower serum albumin, older age, higher serum phosphorus levels, diabetes mellitus, and significant heart failure, a risk factor influencing mortality that differs from the conclusions of published articles was higher baseline eGFR [24, 50, 51]. A more plausible explanation might be that patients presenting with higher GFR were sicker at the initiation of USPD and might have had more severe malnutrition or heart failure present, which led to a poorer prognosis for the patients.

There were several limitations in our study. First, there was an inherent risk of bias given the retrospective nature of our study. For instance, the cardiac function grading of patients could only be accessed by reviewing medical records, which is less objective. In addition, other possible confounders, such as body weight, blood pressure, C-reactive protein levels, degree of muscle loss, and assessment of frailty, were excluded due to the difficulty of obtaining them retrospectively or excessive missingness. Second, we did not consider changes in indicators occurring during follow-up but only included laboratory indicators obtained before treatment started. Third, we enrolled patients who received USPD therapy before December 2019, while follow-up was completed in June 2020. Some patients were followed for a relatively short duration, which might cause bias in the results of mortality and cause of death. Hence, a large-scale prospective study and the development of models to more accurately predict mortality in different periods in older people treated with USPD should be conducted in the future.

## Conclusion

The study observed that the first six months after receiving USPD was the peak mortality period for older patients with ESRD, especially the first month. In particular, the risk of cardiovascular death requires careful vigilance by clinicians. NYHA class III–IV cardiac function and higher WBC count were risk factors associated with death within six months, whereas NYHA class III–IV cardiac function, lower WBC count, lower serum potassium levels, and higher serum calcium levels were risk factors associated with death after six months of USPD. Our study demonstrates that targeted interventions are necessary to reduce mortality in elderly patients receiving USPD at different treatment periods.

## Data Availability

The datasets used and analyzed during the current study are available from the corresponding author on reasonable request with permission of the Second Bethune Hospital of Jilin University, the First Bethune Hospital of Jilin University-the Eastern Division, Xing’an League People’s Hospital, Jilin province FAW General Hospital and Jilin Central Hospital.

## Abbreviations

ESRD: end-stage renal disease
PD: peritoneal dialysis
USPD: urgent-start peritoneal dialysis
USHD: urgent-start hemodialysis
NYHA: New York Heart Association
WBC: white blood cell
APD: automated peritoneal dialysis
CAPD: continuous ambulatory peritoneal dialysis
CVD: cardiovascular disease
Hb: hemoglobin
K: potassium
eGFR: estimated glomerular filtration
Ca: calcium
P: phosphorus
HR: hazard ratio
CI: confidence interval
